# Recent Advances in Applications of Cellulose Derivatives-Based Composite Membranes with Hydroxyapatite

**DOI:** 10.3390/ma13112481

**Published:** 2020-05-29

**Authors:** Madalina Oprea, Stefan Ioan Voicu

**Affiliations:** 1 National Institute for Research and Development in Chemistry and Petrochemistry ICECHIM, Splaiul Independentei 202, 060021 Bucharest, Romania; madalinna.calarasu@gmail.com; 2 Faculty of Applied Chemistry and Materials Science, University Politehnica of Bucharest, Gheorghe Polizu 1-7, 011061 Bucharest, Romania; 3 Advanced Polymer Materials Group, Faculty of Applied Chemistry and Material Science, University Polytehnica of Bucharest, Gheorghe Polizu 1-7, 011061 Bucharest, Romania

**Keywords:** membrane, cellulose, hydroxyapatite, water purification, tissue engineering

## Abstract

The development of novel polymeric composites based on cellulose derivatives and hydroxyapatite represents a fascinating and challenging research topic in membranes science and technology. Cellulose-based materials are a viable alternative to synthetic polymers due to their favorable physico-chemical and biological characteristics. They are also an appropriate organic matrix for the incorporation of hydroxyapatite particles, inter and intramolecular hydrogen bonds, as well as electrostatic interactions being formed between the functional groups on the polymeric chains surface and the inorganic filler. The current review presents an overview on the main application fields of cellulose derivatives/hydroxyapatite composite membranes. Considering the versatility of hydroxyapatite particles, the hybrid materials offer favorable prospects for applications in water purification, tissue engineering, drug delivery, and hemodialysis. The preparation technique and the chemical composition have a big influence on the final membrane properties. The well-established membrane fabrication methods such as phase inversion, electrospinning, or gradual electrostatic assembly are discussed, together with the various strategies employed to obtain a homogenous dispersion of the inorganic particles in the polymeric matrix. Finally, the main conclusions and the future directions regarding the preparation and applications of cellulose derivatives/hydroxyapatite composite membranes are presented.

## 1. Introduction

Among the functional materials currently known, membranes possess a unique characteristic and that is selectivity [1]. Another aspect is represented by the fact that they were the first functional materials known on earth—the membrane of the first unicellular organism [2]. Polymeric membranes were manufactured on a wide scale as filtering materials after the Second World War from the practical necessity of obtaining drinkable water from affected or contaminated natural sources. The first polymer applied industrially to obtain these membranes was cellulose nitrate, an explosive powder used to fabricate bombs and as a consequence, available in large quantities. Membrane technology was constituted in an individual scientific field starting with the research conducted by Loeb and Sourirajan that explained the formation mechanism of asymmetric membranes and the coagulation phenomenon of a polymer from concentrated solution in the presence of a non-solvent [3]. Once the formation mechanism of polymeric membranes was established and understood, more and more materials were developed for different practical applications such as gas separation [4,5,6], protein concentration [7,8,9], heavy metals separation [10], and removal of environmental pollutants [11,12,13]. As time passed, beyond their primary role as filtering materials used to obtain drinking water, membranes were employed in more and more advanced applications, the field with the most advanced performance demands being biomedical engineering. To fulfill the requests of such applications, composite membranes were developed by incorporating nanostructured inorganic fillers in the polymeric matrix thus resulting in a synergistic performance by combining the advantages of organic and inorganic materials [14]. One of the first niche domains targeted the obtainment of hemodialysis membranes [15]. Hemodialysis is the membrane process designed for patients with chronic kidney disease [16] and it is used to substitute kidney functions once every two days. The main chemical species that are separated during this process are urea, uric acid, and excess of creatinine and salts from the human body [17]. Other biomedical applications based on the extracorporeal blood circulation is the artificial lung used especially during open-heart surgeries [18,19] or experimental studies for an artificial liver based on composite membranes functionalized with porcine hepatocytes [20].

Due to the porosity and semi-permeable properties, polymeric membranes are also used in the development of controlled drug delivery devices [21], in an attempt to avoid the toxic effects of high quantities of pharmaceutically active substances on the human body. Another increasingly researched field of application is the one of osseointegration membranes that are placed at the interface between a metallic implant and the bone, with the purpose of favoring the pre-osteoblasts growth and spreading, in order to integrate the implant into the host bone tissue [22,23,24]. These latter membranes are based especially on composites with hydroxyapatite. Hydroxyapatite is one of the natural bone components, the so called “soft component”. It can be used as such, to obtain composites [25,26] or with the addition of other elements, like silver, to obtain hydroxyapatite with antibacterial properties [27]. Hydroxyapatite also has a high synthesis versatility because it can be of animal [28,29] or synthetic origin [30]. The present review desires to offer the reader an overview on the recent progress made in the domain of composite membranes based on cellulose derivatives and hydroxyapatite. Why these two components? They both present the great advantage of originating from natural sources, which is associated with biocompatibility of the resulting composites. More than that, once inserted into the human body, in the case of tissue engineering scaffolds, cellulose presents the remarkable property of bioresorbability, with only glucose molecules resulting after its hydrolysis and degradation. Cellulose derivatives-based membranes for various applications such as water purification, tissue engineering, osseointegration, drug delivery, and hemodialysis will be presented, all having in common hydroxyapatite as a filler agent.

Pure cellulose is frequently turned into cellulose derivatives to overcome the drawbacks related to its poor solubility in common organic solvents [31]. Due to the low cost and widespread availability of cellulose [32], cellulose acetate (CA) and carboxymethylcellulose (CMC) particularly are among the most used cellulose-based matrices for the incorporation of inorganic hydroxyapatite particles in order to obtain hybrid composite membranes with improved physico-chemical and biological characteristics.

Cellulose acetate is a cellulose ester formed by partial or full acetylation of the free hydroxyl groups in the anhydroglucose unit. Depending on the acetyl content that usually ranges between 29% and 48%, mono-, di-, and triacetate can be differentiated [33]. The ability of facile processing by various techniques and its broad range of applications make cellulose acetate the most commonly synthesized cellulose derivative worldwide, the global production of CA from biomass being projected by Global Industry Analysis to be 751.1 thousand metric tons until 2024 [34]. The classic cellulose acetylation process is based on the reaction between wood or cotton pulp with acetic anhydride as the acetylation agent and sulfuric acid as catalyst in an acetic acid reaction media [35]. Currently, this approach is used industrially but more and more research is being conducted on the use of agro-industrial residues and environmentally friendly synthesis routes that involve replacing the sulfuric and acetic acids by eco-friendly reagents [36]. Cellulose acetate is employed for the production of a variety of consumer goods including textiles, photographic films, personal hygiene products, and cigarette filters [37] but its resistance to the action of chemical agents, good thermal stability, flexibility, and mechanical strength [38], coupled with low fouling susceptibility and a hydrophilic nature [39], recommend this polymer especially for the production of membranes, with applications in industrial and biomedical processes (e.g., adsorption, separation, catalysis, biosensing, drug delivery, or tissue regeneration). Cellulose acetate was used in the purification process of contaminated resources of natural gas starting with the mid 1980s, when several companies applied dried CA membranes for CO_2_/CH_4_ natural gas separations. The hydrophilic nature of cellulose acetate makes the membranes suitable for assisting chemical and biochemical reactions as well as for the removal of polar compounds or specific organic–organic separations using pervaporation. The separation of helium particularly is of great interest in the natural gas purification process, due to its high added value. Asymmetric CA membranes presented an acceptable permeability for He and a good He/N_2_ and He/CH_4_ selectivity, being considered economically feasible for usage in three stage membrane processes with recycle streams [40]. Carboxymethyl cellulose, one of the most important cellulose ethers, is synthesized by treating alkali cellulose with monochloroacetic acid or sodium monochloro-acetate in an aqueous sodium hydroxide (NaOH) medium [41]. Wood residues, cotton linters, paper sludge, and agricultural waste biomass such as orange peels, corncobs, sugarcane bagasse, rice, or corn husks were used so far as cellulose sources for the preparation of carboxymethylcellulose [42]. As the reaction takes place, the hydroxyl groups in the cellulose backbone are replaced with carboxymethyl groups in the C6 > C3 > C2 order [43]. The chemical structure obtained following etherification is responsible for the unique properties of carboxymethylcellulose, such as water solubility, non-toxicity, biodegradability, transparency, and good film forming ability [44]. Owing to these characteristics, carboxymethylcellulose is already applied in the food, pharmaceutical, and daily-use chemical industries as an emulsifier, thickener, and a flocculating or chelating agent [45,46]. Currently, carboxymethylcellulose based materials are investigated for biomedical applications such as tissue engineering [47] and drug delivery [48] mainly in the shape of hydrogels, membranes, and nanoparticles [49].

Some of the most popular membrane manufacturing techniques include electrospinning, gradual electrostatic assembly, and phase inversion by immersion precipitation or solvent evaporation.

Phase inversion is a popular method for the preparation of cellulose acetate membranes. The first step of this process consists in the dissolution of the polymer in an appropriate solvent, such as hexafluoro-2-propanol [50], formic acid [51], N, N-dimethylformamide [52], acetone [53,54,55], or a mixed solvent system of the latter two [56], to obtain a homogenous polymeric solution. Afterwards, the obtained solution is cast on a glass plate and submerged in a coagulation bath containing a non-solvent, usually distilled water. The penetration of the solvent into the non-solvent and non-solvent into the polymeric solution cause demixing and polymer precipitation with the formation of a membrane with an asymmetric structure, composed in most cases of a thin film top layer also called “skin”, a support porous substructure, and a bottom layer [54,57].

Gradual electrostatic assembly is based on the spontaneous interactions between two oppositely charged polysaccharides, mixed in an aqueous solution. The electrostatic interactions are followed by polymeric chain entanglement and hydrogen bond formation, this resulting in a polyelectrolyte complex membrane. Due to its anionic nature, carboxymethylcellulose was used in combination with cationic polymers such as chitosan (CS) to prepare such composites [58].

Cellulose acetate was among the first electrospun polymers [59]; it is considered suitable for electrospinning because it can be easily dissolved in common organic solvents and maintains high mechanical strength during membrane fabrication [60]. To date, there are no studies that report the effective electrospinning of carboxymethylcellulose without the addition of another polymer; nonetheless, blends of CMC with polyvinylpyrrolidone (PVP) [61], polyvinyl alcohol (PVA) [62], or polyethylene oxide (PEO) [63,64] were successfully electrospun into nanofibrous membranes with applications especially in the biomedical area. The electrospinning experiments usually take place at room temperature under normal atmospheric conditions. The device consists of three major parts: high voltage power supply, feeding nozzle or spinneret, and a grounded collecting plate (metal plate or rotating drum). The polymeric solution is inserted into a capillary tube connected to a feeding nozzle. A high voltage source is used to inject a certain polarity charge into the polymeric solution or melt. When the electric field reaches a critical value the repulsive electrical forces surpass the surface tension forces at the tip of the nozzle and the solution is accelerated towards the opposite polarity collector. The solvent is evaporated and polymeric fibers are formed [65]. Solution viscosity, polymer concentration, and molecular weight are important factors that influence the electrospinning process and an optimal balance between them must be achieved in order to generate uniform fibers. Due to their unique properties, electrospun fibers have been successfully applied in various domains such as environmental engineering, pharmaceutics, optoelectronics, biomedicine, and biotechnology [66].

**Figure 1:**
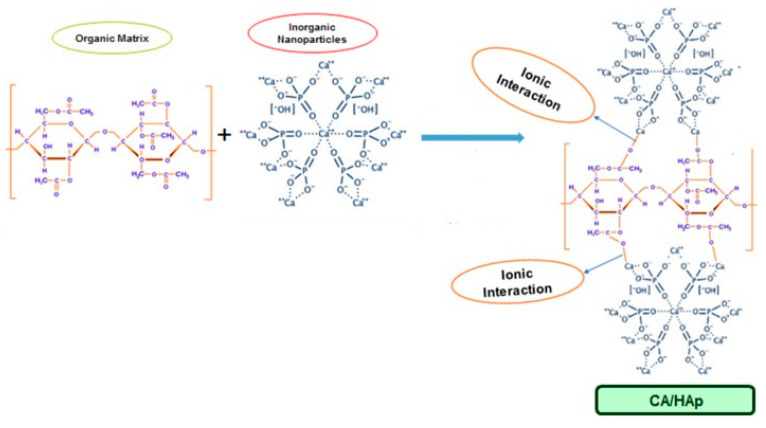
Proposed formation mechanism of cellulose acetate/hydroxyapatite hybrid composites (reproduced with permission from Ref. [60]).

Lately, an increased research interest was directed towards the utilization of cellulose derivatives/hydroxyapatite composite membranes in environmental or biomedical engineering (e.g., water purification, bone tissue regeneration, wound healing, controlled drug delivery). It was found that there is a good compatibility between cellulose-based matrices and hydroxyapatite, the inorganic particles interacting with the organic component by inter and intramolecular hydrogen bonds, and also by ion-dipole forces formed between the calcium ions of HA and the functional groups on the cellulose derivatives surface [60]. For example, in the case of CA, the positively charged calcium ions bind with the negatively charged carboxylate groups and hydrogen bonds are formed between the hydroxyl groups of cellulose acetate and hydroxyapatite (Figure 1).

It is demonstrated that hydroxyapatite (HA) particles are good mineral adsorbers and have the capacity to bind divalent heavy metal ions, hence they were researched for the removal of harmful substances in drinking water [53]. Hydroxyapatite particles can also be used as additives for cellulose acetate to improve the morphology and properties of the membranes, this resulting in a better separation performance and higher water flux values [54]. More than that they could act as a catalyst for the precipitation process involved in phase inversion, by increasing the viscosity of the solution [39,57]. The most common synthesis methods for membranes are phase inversion with two possible variants—precipitation in a non-solvent and solvent evaporation. The most versatile is the precipitation of the membrane in a non-solvent. The non-solvent flow through solution polymer film will determine the porosity and morphology depending on several parameters—viscosity, temperature, and miscibility with the polymer solvent. These properties directly influence the speed of membrane formation—the higher the formation speed, the smaller the pore diameter with a high distribution of pores at the surface. A slow process of membrane coagulation will lead to pores with a large diameter and a lower distribution of pores on the surface. Each possibility is preferred depending on the application of the synthesized membrane. The presence of hydroxyapatite in a polymer solution acts in two ways—firstly it will increase the viscosity of the solution and second it will influence the speed of non-solvent through the polymer solution film. The first influence is always higher, so from a more viscous initial solution, membranes with a decreased diameter of pores will be obtained. This can be translated into a more efficient separation. More than that, hydroxyapatite particles itself, in the structure of the membrane, participate in the separation process due to their porosity and ability to retain small chemical species like cations, pesticides, and dyes.

An arising issue is represented by the aggregation tendency of the inorganic particles but various strategies such as ultrasound-assisted mixing [52], surfactants addition [54], or modification of the hydroxyapatite surface [56] are investigated for an improved dispersion. The aggregation tendency of hydroxyapatite particles in aqueous media was successfully overcome by using electrospinning as membrane fabrication technique. Electrospun membranes have some advantages over phase inversion membranes in terms of volume and aspect ratio, specific area, and porosity, but, according to a recent study, HA concentrations higher than 3 wt/v% generated a “beads on a string” morphology in the case of CA solutions (15 wt/v%) in a mixed solvent system of acetone and N,N-dimethylformamide (DMF) [60]. Therefore, the inorganic filler concentration must be carefully chosen in order to obtain smooth nanofibers. Due to its biocompatibility, bioactivity, and osteoconductive properties, hydroxyapatite distinguished itself among materials used for bone tissue regeneration. However, the direct use of hydroxyapatite in such applications was associated with poor mechanical and chemical stability especially in the case of synthetic particles [67]. Therefore hybrid polymer/hydroxyapatite composites were developed, natural polymers such as collagen [68,69], polylactic acid [24,70], chitosan [71,72], and cellulose [73] being preferred, instead of synthetic ones [74], for the preparation of novel composite materials with superior bioactivity compared to pure components.

## 2. Applications of Cellulose Derivatives/Hydroxyapatite Composite Membranes

### 2.1. Water Purification

**Figure 2:**
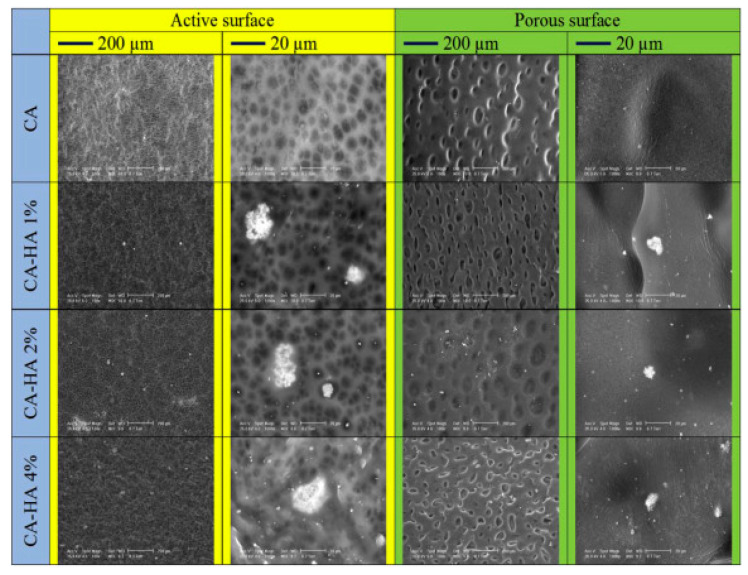
SEM images of the cellulose acetate/hydroxyapatite membranes prepared by immersion precipitation (reproduced with permission from Ref. [52]).

Membrane technology received great attention and was widely applied in the process of separation of toxic compounds from water. Various studies were focused on the improvement of cellulose derivatives/hydroxyapatite composite membranes morpho-structural characteristics in order to ensure an increased water flow without affecting their filtration ability. For example, Pandele et al. prepared cellulose acetate/hydroxyapatite membranes by immersion precipitation and used ultrasound assisted mixing to achieve a homogenous filler dispersion throughout the organic phase. The membranes presented a dense surface with embedded micro-structured hydroxyapatite crystals and conical shaped pores on the bottom side (Figure 2) [52]. In this case, scanning electron microscopy presents surface aspect changes and the emergence of hydroxyapatite crystals in the composite membranes, both on the active and on the porous surface of the membrane. On the active surface, a slight decrease of the average diameter of the pores and shape in the case of composite membranes was observed compared to membranes made of neat cellulose acetate (explained by weak chemical interaction between the acetate groups of the polymer and the phosphate groups of HA). In such cases these interactions can lead to internal defects in the membrane structure. A difference in pore diameter on active surface and porous surface of the membranes can also be easily observed. This can be explained by the precipitation of the polymer film into a non-solvent which generate an asymmetric structure of the membrane with a conical shape of pores in cross section of the membrane [2,12]. Water flows increased by increasing the concentration of hydroxyapatite, ranging from 8.29 L/m^2^h for the CA membrane to 20.96, 23.25, or 26.73 L/m^2^h for the composite membranes with 1, 2, and 4 wt. % HA due to the uniform addition of hydroxyapatite particles within the membrane structure.

**Figure 3:**
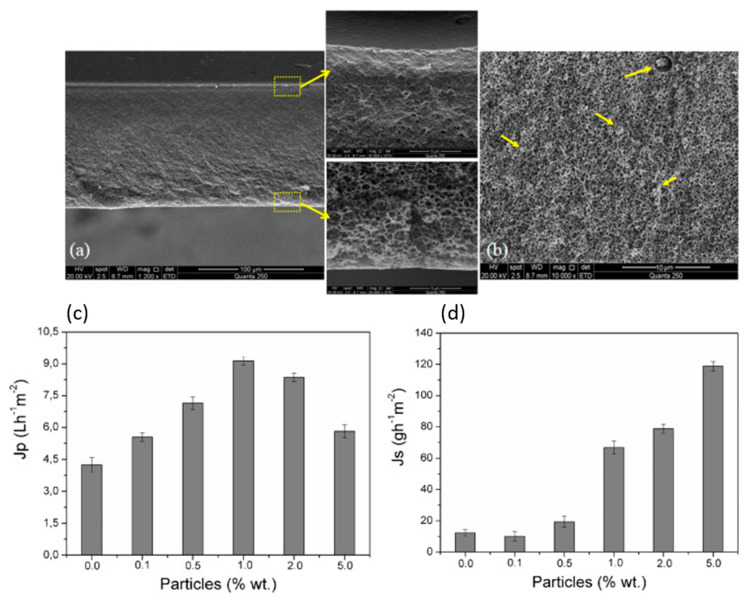
SEM images of the anisotropic cellulose acetate/hydroxyapatite membranes with details of top and bottom layers (**a**); particle agglomerates observed in the cross-section of the membrane containing 5 wt. % hydroxyapatite (HA) (**b**); water (**c**); and salt flux (**d**) of the composite membranes obtained during permeation tests (reproduced with permission from Ref. [56]).

Despite the sonication treatment, high amounts of filler cannot be dispersed uniformly throughout the membrane and hydroxyapatite clusters with high concentrations are formed. Another dispersion improvement strategy was reported by Ohland et al. and it is represented by the functionalization of the hydroxyapatite particles in a methanol plasma atmosphere, prior to their ultrasound-assisted incorporation in the polymeric solution. X-Ray Photoelectron Spectroscopy (XPS) analysis of the functionalized hydroxyapatite revealed an increase in the oxygen atomic percentage and also in the oxygen/carbon ratio, this indicating that the methanol plasma precursor successfully provided hydroxyl groups that were further transferred to the particles surface, this increasing their hydrophilicity and compatibility with the polymeric matrix. The composite membranes prepared using phase inversion with partial solvent evaporation before precipitation presented an anisotropic morphology (Figure 3a). The plasma-treated hydroxyapatite particles were well dispersed, aggregates being observed only at 5 wt. % HA concentrations and higher (Figure 3b). The incorporation of the functionalized particles in the cellulose acetate matrix improved the membrane affinity and lead to an increased water and salt flux in forward and reverse osmosis permeation tests (Figure 3c,d) [56]. In another study conducted by the same research group, the plasma functionalized hydroxyapatite particles were incorporated in both layers of a dual-structured membrane composed of a porous cellulose acetate support obtained by phase inversion and a selective polyamide layer synthesized over the porous support by interfacial polymerization. The addition of the functionalized hydroxyapatite particles in the cellulose acetate support improved the hydrophilicity and reduced internal concentration polarization, which further increased water flux in forward osmosis without affecting the reverse salt flux. More than that, the incorporation of functionalized hydroxyapatite in the selective polyamide layer reorganized the polymeric chains and improved its affinity with water, this resulting in lower diffusion resistance and improvement of water permeability in reverse and forward osmosis [75].

High performance ultrafiltration membranes based on cellulose acetate and nano-hydroxyapatite were prepared using the surfactant assisted phase inversion method to prevent particle agglomeration. The nano-powder was first dispersed in 12-hydroxystearic acid, an amphiphilic surfactant, and then mixed homogenously with the polymeric solution, acetone being used as solvent in both cases. The filler loading percentage was varied between 10 and 30 wt. %, the best results being obtained for the membrane containing 30 wt. % hydroxyapatite. The higher water flux values and the increase in salt rejection capability of the composite membranes were attributed to the formation of hydrophilic nano-pores in the top layer and in the pore walls of the middle layer, coupled with the negative surface charge. These characteristics are thought to also be involved in the modification of the fouling behavior [54]. Overall, the addition of an amphiphilic surfactant during the membrane preparation process was shown to be an effective way to prevent the hydroxyapatite particle aggregation and allowed the effective incorporation of a high filler percentage in the organic matrix. The membranes obtained by this method presented superior performances in terms of water flux values (34.96 L/m^2^ h bar) and fouling resistance compared to previously reported studies [52,56,75].

**Figure 4:**
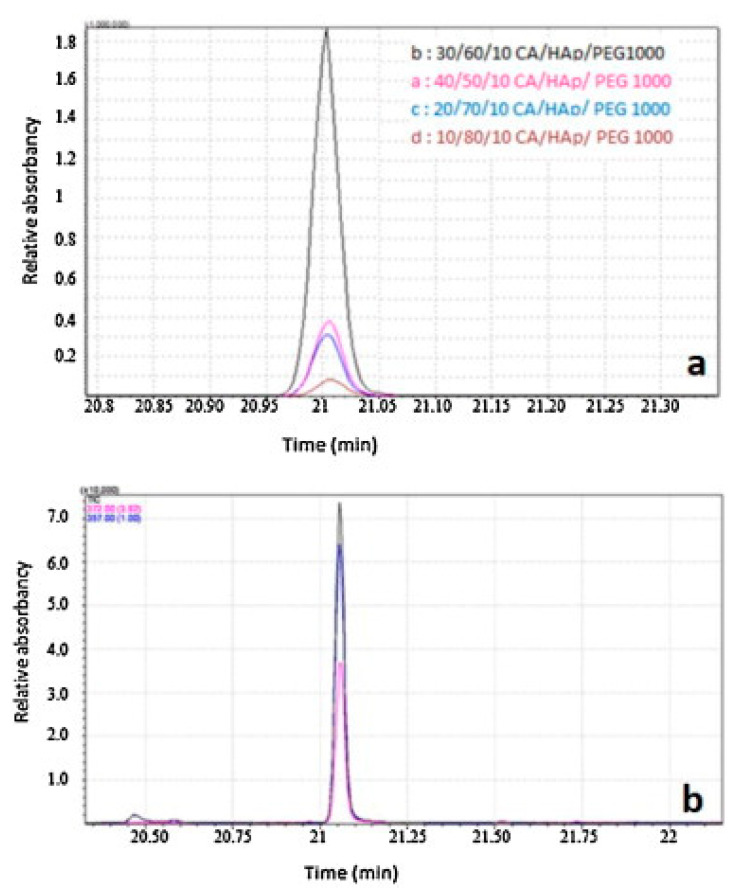
Chromatograms of bisphenol-A desorbed from cellulose acetate/hydroxyapatite/poly(ethylene glycol) (CA/HA/PEG) composite membranes (**a**) and commercial Florisil (**b**) (reproduced with permission from Ref. [53]).

Azzaoui et al. used polyethylene glycol (PEG) 1000 as surfactant, during the hydroxyapatite synthesis stage, to obtain highly dispersed particles that were further incorporated into cellulose acetate [53] or hydroxyethyl cellulose acetate (HECA) [76] matrices. The hybrid membranes were prepared using phase inversion by controlled evaporation, a procedure also known as solvent casting. Morphology studies showed a good compatibility between both of the two organic matrices (CA and HECA) and hydroxyapatite particles. The thermal stability of the composites increased proportionally with the loading of HA but was slightly lower compared to the neat polymers. For example, the decomposition temperature of HECA/HA/PEG ranged between 269–353 °C while neat HECA decomposed between 280–375 °C, respectively, CA/HA/PEG composites decomposed between 355–375 °C while neat CA membranes presented a maximum decomposition temperature of ~376.88 °C. Nuclear Magnetic Resonance (NMR) spectra revealed the presence of hydrogen bonding between carbon–oxygen groups in HECA and the hydroxyl groups on the inorganic particles surface. The CA/HA/PEG membranes were further tested for their ability to separate toxic compounds from water. They presented recovery rates of adsorbed bisphenol-A, a toxic and carcinogenic organic compound from polluted water, comparable with the commercial Florisil^®®^ adsorbent for chromatography, notably in the case of the 30/60/10 CA/HA/PEG composite (Figure 4). The study concluded that thin CA/HA/PEG composite membranes coupled with gas chromatography-mass spectrometry (GC–MS) analysis could be successfully used for the retention and detection of potential toxic compounds in water such as bisphenol-A [53].

Other examples of toxic compounds in water include heavy metals, pesticides, polychlorinated biphenyls, and inorganic and organic poisons [77]. Nanofibrillated cellulose acetate/hydroxyapatite membranes were tested for the adsorption of Fe (III) and Pb (II) ions in wastewater. The experimental adsorption procedures, conducted using solutions of 10 ppm metal ion concentration, showed that CA/HA nanofibers recorded a 100% removal efficiency for Pb (II) and 95.5% for Fe (III) while neat CA nanofibers recorded only a 19.3% and 22.6% removal of Pb (II) and Fe (III) ions respectively. The adsorption potential was highly increased by the addition of hydroxyapatite due to the chemical and physical affinity between the positively charged metal ions and the functional groups on the filler particles surface. Porosity also played an important role in the improvement of adsorption rates, the heavy metal ions hydroxide precipitates being retained not only on the surface but also in the depth of channels and pores of the membranes [60]. Similar results were obtained in the case of cellulose pulp/carboxymethyl cellulose/nano-hydroxyapatite composite paper sheets tested for the adsorption of iron, lead, and cadmium ions in polluted water [78].

Carboxymethylcellulose was also used in combination with hydroxyapatite to prepare biodegradable nanocomposite membranes for the removal of bisphenol-A from water. The membranes were fabricated by two different methods, the conventional solution casting and the double decomposition procedure. Double decomposition is a common method used for the synthesis of hydroxyapatite. It consists of the decomposition of both calcium and phosphate sources in an aqueous solution to form hydroxyapatite precipitates [79]. In this case Ca(NO_3_)^.^4H_2_0 and (NH_4_)_2_HPO_4_ were precipitated in an aqueous CMC solution and lysine, an essential amino acid with antibacterial activity due to its amine group [80], was used as a plasticizer. The membrane fabricated by double decomposition had superior characteristics in terms of filler dispersion and flexibility. Due to the improved dispersion of the inorganic particles in the polymeric matrix, the double decomposition membrane kept its transparency after the addition of HA, while the casted one became opaque. The bisphenol-A extraction efficiency was evaluated using gas chromatography coupled with mass spectrometry. The highest recovery rate was recorded for the membranes obtained by double decomposition containing a mass ration of 20:70:10 CMC/HA/lysine. These membranes also presented good antifungal and antimicrobial properties against *Candida albicanis*, Gram-positive *Bacillus subtillus* and Gram-negative *Escherichia coli*, this indicating their multi-functionality and potential to be used for both water purification and biomedical applications [44].

### 2.2. Bone Tissue Engineering

**Figure 5:**
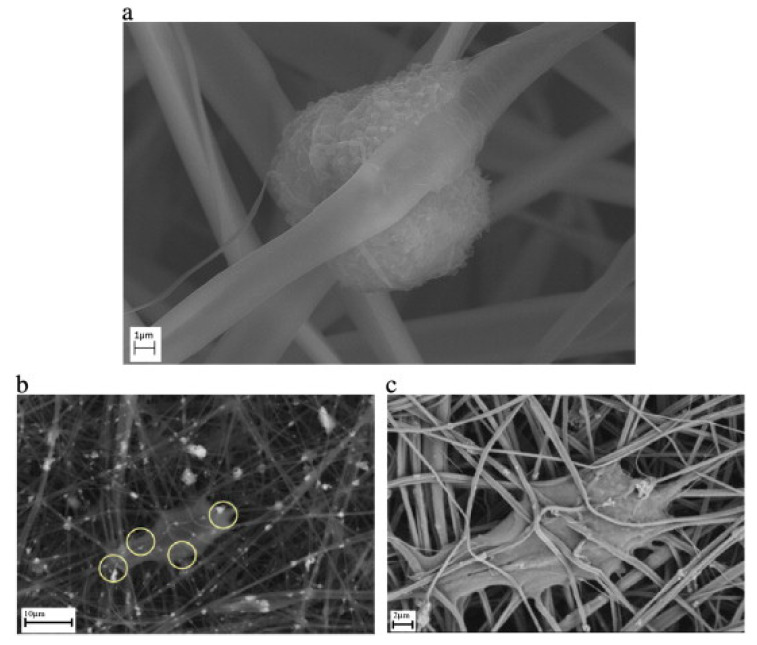
Differences between osteoblast cells grown on neat cellulose acetate fibers (**a**) and composite cellulose acetate/nano-hydoxyapatite fibers at low (**b**) and high (**c**) magnification (reproduced with permission from Ref. [81]).

The use of cellulose derivatives/hydroxyapatite composite membranes as artificial scaffolds for in vitro regeneration of bone tissue was reported in several studies. Osteoblasts are anchorage dependent cells whose attachment on scaffolds depends on the surface area and porosity. Electrospinning cellulose acetate results in biocompatible membranes, with appropriate structural characteristics for cellular attachment and migration. The thin fibers serve as attachment sites for cells while the interconnected porosity allows the cellular migration inside the membrane. Studies showed that the fibrous cellulose acetate membranes are very flexible and the pores can dynamically expand to accommodate growing cells (Figure 5a). The bioactivity is ensured by the hydroxyapatite particles that are involved in both initial adsorption of adhesion proteins such as fibronectin and vitronectin, that will further serve as anchorage sites for osteoblasts (Figure 5b,c) and in cellular differentiation and promotion of calcium phosphates deposition [81].

Surface modifications via oxygen plasma or alkaline treatments were reported for the improvement of the membranes biological activity by mechanisms such as migration of the hydroxyapatite filler towards the fiber surface or the increase in hydrophilicity and deacetylation of cellulose acetate [82]. According to a study conducted by Tao et al., electrospun cellulose acetate/silk fibroin/hydroxyapatite membranes are an efficient carrier for the sustained release of bone morphogenetic protein-2 (BMP-2). Silk fibroin contains ligands that promote cell adhesion, migration, and proliferation and can also support the differentiation of mesenchymal stem cells into osteoblasts. BMP-2 is a growth factor that plays an important role during the development and regeneration of bone tissue. Coaxial electrospinning was employed for the membranes preparation, the fibers consisting in a cellulose acetate core and a silk fibroin shell in which nano-hydroxyapatite and BMP-2 were included. The presence of hydroxyapatite and the controlled release of BMP-2 simulated the proliferation and osteogenic differentiation of bone marrow mesenchymal stem cells seeded on the fibrous membranes and also the cell—mediated calcium accumulation. The fibrous composites were implanted directly on a bone defect site to study their influence on the tissue regeneration in vivo. It was observed that the presence of the material significantly increased the rate of novel bone tissue formation in rat cranial defects after 12 weeks of implantation [83].

**Figure 6:**
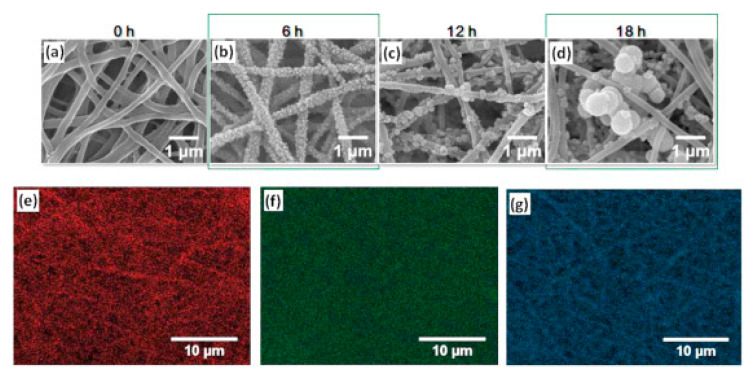
SEM images of hydroxyapatite coated sodium carboxymethylcellulose nanofibers mineralized for 0 h (**a**), 6 h (**b**), 12 h (**c**), 18 h (**d**), and elemental mapping images of the NaCMC/HA nanofibers mineralized for 6 h: carbon map (**e**), calcium map (**f**), and phosphorus map (**g**) (reproduced with permission from Ref. [86]).

Natural bone is an anisotropic material composed of cells, a collagen matrix, and calcium phosphates in the form of hydroxyapatite. Inspired by the constituents of the natural bone, hybrid membranes based on cellulose acetate, hydroxyapatite, and cellulose microfibers were studied as materials for bone tissue engineering. Cellulose microfibers extracted from raw cotton were selected as reinforcing agents for the cellulose acetate matrix due to their resemblance with collagen. The microfibers were coated with hydroxyapatite by immersion in simulated body fluid (SBF) prior to their dispersion in the cellulose acetate solution using cetrimonium bromide (CTAB) as surfactant. The thin membranes obtained after solvent evaporation presented a porous morphology, favorable for osseointegration and were flexible enough to be rolled and stacked, if required, to increase their mechanical strength and anisotropy [84]. Hydroxyapatite deposited from SBF was also used for the coating of cellulose acetate/polyvinylpyrrolidone (PVP) electrospun membranes. The immiscibility of the two polymers coupled with the low surface tension of PVP lead to a phase separation during the electrospinning process, this causing the formation of a dual fiber structure composed of an inner CA core and an outer PVP discontinuous shell. As the PVP content increased, the uniform cylindrical fibers morphology was gradually lost, flat fibers with multiple surface grooves being obtained. However, the composite fibers provided more favorable conditions for biomineralization, the grooves and cavities facilitating the hydroxyapatite particles deposition and crystals growth. Additionally, the thermal stability was improved due to the formation of hydrogen bonds between CA and PVP [85]. Yamaguchi et al. also reported a successful method for the fabrication of hydroxyapatite coated cellulose-based membranes by biomineralization in SBF. Cellulose acetate nanofibers were deacetylated, then treated with sodium hydroxide and subsequently carboxymethylated using a sodium carbonate/chloroacetic acid mixture to obtain sodium carboxymethylcellulose (NaCMC) nanofibers. It was noticed that the NaOH concentration, biomineralization time, and bicarbonate ion (HCO_3_^−^) density had an important influence on the hydroxyapatite crystals formation and growth. At higher NaOH and HCO_3_^−^ concentrations, a large number of fine hydroxyapatite crystals were formed on the nanofibers probably due to the increased substitution degree of the carboxyl groups and the intermolecular interactions between HA particles and HCO_3_^−^ ions. A longer biomineralization time lead to crystal growth and formation of aggregates in the hollow spots between the nanofibers (Figure 6). A mineralization time of 6 h was found to be optimal for the proliferation of MC3T3-E1 cells, the fine attachment and homogenous dispersion of hydroxyapatite on the NaCMC nanofibers favoring cellular anchorage and development [86].

Other cellulose based matrices reported in literature for biomimetic mineralization include composites of ethyl cellulose (EC) and hydroxypropyl cellulose (HPC) with polyacrylic acid (PAA). The composites were soaked in salt solutions containing small amounts of PAA to prevent mineral precipitation and thermostated at 30 °C for 5 days, the solutions being frequently changed to maintain an optimal pH value (8.5–9). The mineralization rate was proportional to the content of PAA that served as an ion adsorbent. XRD and EDS analysis revealed that the minerals were incorporated in the polymeric matrix in the form of amorphous calcium phosphate, hydroxyapatite, and PAA-Ca^2+^ complexes. The thermal and mechanical properties were remarkably improved after mineralization, especially in the case of EC/PAA composites that presented a higher percentage of deposited inorganic compounds [87].

**Figure 7:**
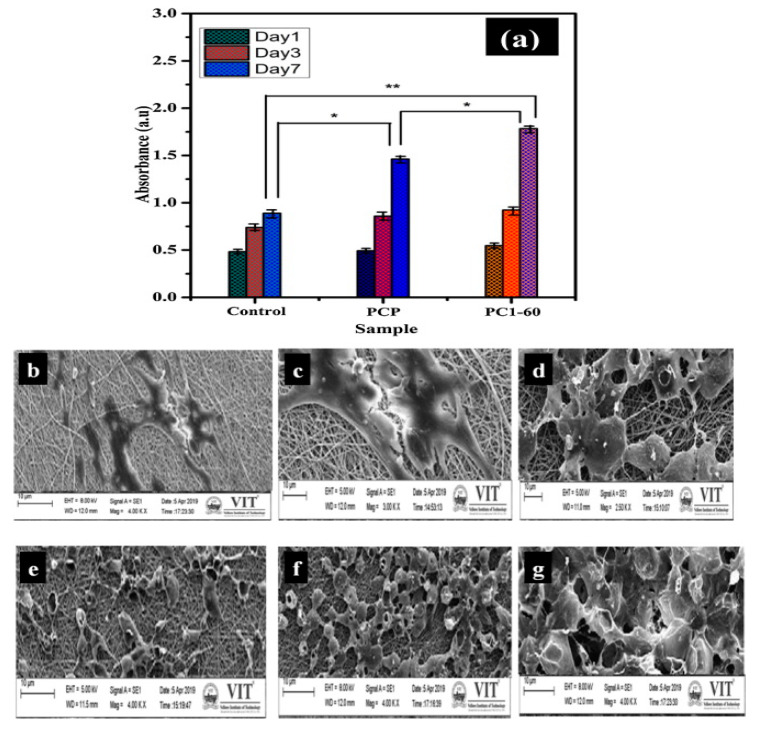
MTT assay results for HOS cells seeded on CA/PVP (PCP) and CA/PVP/HA 0.1M Zn–Mg (PC1-60) membranes on the 1st, 3rd, and 7th days (**a**); microscopy images showing cellular growth (**b**–**d**) and attachment (**e**–**g**) on PCP and PC1-60 membranes (reproduced with permission from Ref. [61]). * Significant difference from all in day 3 (*p* < 0.5). ** Significant difference from all in day 7 (*p* < 0.5).

Recent studies showed that carboxymethylcellulose stimulates adhesion, spreading, and migration of mouse fibroblasts in vitro and also has the potential to induce osteogenic differentiation. These findings support its use in the fabrication of materials for bone tissue regeneration [88]. Carboxymethylcellulose blends with polyvinyl pyrrolidone (PVP) and sodium tripolyphosphate as crosslinking agents were reported for the preparation of electrospun nanofibrous membranes with applications in bone tissue engineering. For an improved bioactivity, hydroxyapatite doped with different quantities of zinc and manganese was incorporated in the CMC/PVP solutions prior to the electrospinning process. According to previous studies, the slow release of zinc and manganese ions at the defect site has the ability to enhance novel bone formation and growth around implants and also improves cellular adhesion. The cellular proliferation ability of human osteoblasts (HOS) seeded on the composite membranes was evaluated by 3-[4-5-dimethylthiazole-2-yl]-2,5-diphenyltetrazolium bromide (MTT) assays at different time periods (1, 3, and 7 days). The cellular proliferation and attachment increased proportionally to the culture time, this indicating the non-toxicity of the fibers (Figure 7). The antimicrobial activity and hemocompatibility were also evaluated. The highest zone of microorganism’s growth inhibition being observed for the membrane containing 60 wt. % hydroxyapatite doped with 0.1 M Zn–Mn (PC1-60). This membrane was also the most hemocompatible, with hemolysis values lower than 3% when compared to the other formulations [61].

Polyethylene oxide (PEO) was also found to be an effective additive for the improvement of CMCs electrospinnability, the emergence of hydrogen bonds between the macromolecules of CMC and PEO, combined with the polymeric chains entanglement, resulting in a crosslinked structure which allows the spinning of nanofibers. A detailed study on the influence of the electrospinning parameters on the formation of nanofibers from aqueous mixtures of carboxymethylcellulose/polyethylene oxide/nano-hydroxyapatite was performed by Gasparic et al. It was found that a blend of 7 wt. % CMC and 5 wt. % PEO (CMC:PEO 1:1 wt./wt.), electrospun using a voltage of 60–65 kV and a distance of 150 mm between electrodes, at a relative humidity lower than 50% at 20 °C, produced high quality nanofibrous membranes. The addition of hydroxyapatite nanoparticles broadened the size distribution of the fibers but their diameter remained on the nanoscale. Additionally, the prepared membranes were hydrophobized by impregnation with alkenyl succinic anhydride, to extend their range of applications by making them insoluble in water. Cellular viability results were similar to the ones obtained for commercial collagen/apatite scaffolds. Osteoblasts grown on the hydrophobized membranes presented similar viability but reduced cellular attachment compared to the commercial substrates [64]. Another strategy to improve the properties of CMC was reported by Qi et al. and it consisted in the protonation of CMC nonwoven sheets. To prepare the nonwoven sheets, pre-purified cotton linters were dissolved in cuprammonium and the obtained solution was ejected from the spinning nozzle of a net conveyor. The resultant cellulose nonwoven sheets were then carboxymethylated using a mixture of sodium chloroacetate and sodium hydroxide [49]. For protonation, the sheets were immersed for 1 h in a mixture of nitric acid and methanol. The protonated sheets were then loaded with calcium phosphates by an alternate soaking method in aqueous solutions of CaCl_2_/Tris-HCl and NaH_2_PO_4_ to obtain flexible and easy to handle scaffolds for bone regeneration. XRD spectra showed that the deposited calcium phosphate was a mixed phase of brushite (dicalcium phosphate dihydrate) and hydroxyapatite. The highly soluble brushite could act as a calcium resource and promote mineralization during osteogenesis while hydroxyapatite contributes to osteoconductivity. Further analysis indicated that a higher protonation degree determined a more effective loading due to the suppression of the fibers swelling during the soaking process, this leaving the fiber gaps open for calcium phosphates deposition. Also, the sheet dissolution time can be adjusted according to the desired application by modifying the protonation degree [88].

**Figure 8:**
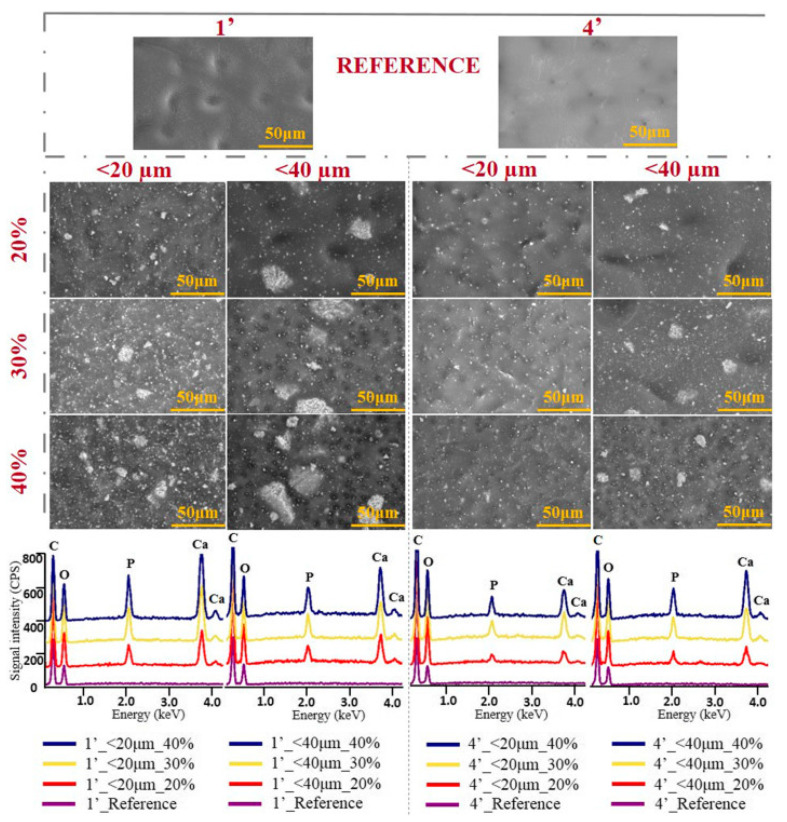
The influence of the three key synthesis parameters—ultrasonic dispersion time, hydroxyapatite particles size, and powder concentration—on the morpho-compositional features of the composite cellulose acetate/hydroxyapatite membranes (reproduced with permission from Ref. [91]).

Guided bone regeneration is a surgical procedure that uses barrier membranes, with or without bone substitution materials, to avoid the interfering of non-osteogenic components in tissue regeneration, prior to the installment of metallic implants. The barrier membrane plays a vital role in the guided bone regeneration process, therefore, the optimization of membrane materials, both in terms of barrier and bioactive properties, represents an important research topic [89]. Composites based on cellulose acetate and hydroxyapatite represent a suitable option for guided bone regeneration as long as an optimal synthesis route is developed to ensure best membrane performances in this field. In a recent study conducted by Dascalu et al., the influence of tree key synthesis parameters—ultrasonic dispersion time, hydroxyapatite particles size, and powder concentration—on the final cellulose acetate/bovine-bone derived hydroxyapatite membrane characteristics was investigated. Two sorts of hydroxyapatite powders (20 µm vs. 40 µm particle size) were added in different concentrations (20%, 30%, and 40%) in cellulose acetate solutions and sonicated for 1 or 4 min. The biocomposite membranes were obtained by immersion precipitation, using distilled water as non-solvent. The variation of the synthesis parameters influenced both surface and bulk characteristics of the membranes. It was determined that a uniform dispersion and a homogenous surface and volume aspect is favored by smaller hydroxyapatite particles sizes and a longer sonication time even at high concentrations (Figure 8). In vitro biocompatibility assays were also performed using mouse pre-osteoblasts (MC3T3-E1) and the results were favorable, all the analyzed membranes eliciting a good cellular response in terms of adhesion and viability [90].

**Figure 9:**
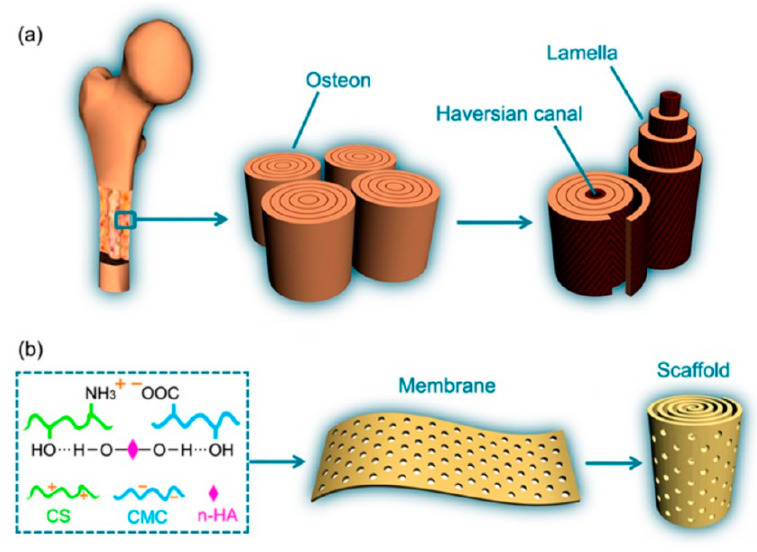
Structural comparison between natural bone (**a**) and the CS/NaCMC/n-HA spiral-cylindrical scaffold (**b**) (reproduced with permission from Ref. [93]).

Carboxymethylcellulose/chitosan blends were also studied in combination with nano-hydroxyapatite (n-HA) for the preparation of membranes with applications in guided bone regeneration. Different techniques were employed for the preparation of such membranes but gradual electrostatic assembly was found to be the most effective for obtaining a uniform, homogenous structure. Jiang et al. studied three types of blending methods to determine which one is optimal for the electrostatic self-assembly of sodium carboxymethyl cellulose (NaCMC) and chitosan (CS) without phase separation or aggregation of the nano-hydroxyapatite particles or polymeric components. Layered casting was used in early studies, more specifically, a certain amount of nano-hydroxyapatite was homogenized into an aqueous NaCMC solution and the mixture was poured in a Petri plate. Afterwards, a chitosan solution in acetic acid was casted slowly on the NaCMC/n-HA wet membrane and the tri-composite material was dried at room temperature and crosslinked with CaCl_2_. However, SEM analysis showed that the chitosan solution did not penetrate into the NaCMC/n-HA liquid membrane and the electrostatic interactions were limited only at the interface between the two phases [91]. To avoid the formation of this double-layered structure, layered casting was replaced with solution blending. The process consisted of the preparation of two different solutions, CS in acetic acid and NaCMC/n-HA slurry in distilled water, which were further mixed together under continuous stirring. The ternary blending mixture was dried at room temperature, immersed in NaOH 5% solution for 24 h, and then dried again. It was noticed that a membrane cannot be formed using this technique, the organic components having the tendency of agglomeration into macroscopic particles or clusters due to the immediate electrostatic interactions that appear between the cationic groups of CS and anionic groups of NaCMC when the two separate solutions are mixed together. Finally, it was established that the optimal blending method was the incorporation of n-HA slurry and chitosan powder into the aqueous NaCMC solution followed by dropwise addition of acetic acid under intense stirring for the gradual dissolution of the CS particles. The extended chitosan chains connect to the surrounding NaCMC matrix through electrostatic interactions and intermolecular hydrogen bonds forming a polymer composite reinforced with n-HA particles that also form hydrogen bonds with the organic matrix. This homogenous structure provided enhanced hydrostability to the membrane, preventing its progressive collapse into pieces, as observed in the case of the layered casted and solution blended membranes when soaked into water. The nano-hydroxyapatite content influenced the tensile strength and elongation rate, which presented an increasing trend with the n-HA percentage up to 60 wt. %, a sharp decrease being recorded at higher values [58]. This optimized blending technique was used for the preparation of NaCMC/CS/n-HA composites for bone tissue regeneration. The membrane containing 60 wt. % n-HA displayed the highest osteoblast viability and osteocalcin expression during the cellular compatibility tests. For an improved integration with the anisotropic natural bone tissue, the membranes were treated by mechanical perforation with a pore size of 300 µm (1 mm pore-to-pore spacing) and curled in a concentric manner to obtain spiral-cylindrical scaffolds with an osteon-like structure (Figure 9). After rolling, the membranes presented a tensile strength comparable to that of cancellous bone (4.91 MPa) and were therefore considered appropriate to support new bone formation at the site of implantation. The materials were implanted in radial defects models of New Zealand white rabbits and radiopacity was observed in the whole implant area after 12 weeks due to the new bone tissue formation throughout the entire scaffold and its remodeling into cortical bone. Histological analysis of the cross-sections collected from the middle part of the scaffold revealed that the newly formed bone tissue grew along the spiral wall and bone marrow penetrated the entire cylinder forming a medullar cavity in the center, this indicating a successful osseointegration and functional reconstruction facilitated by the spiral-cylindrical architecture. The study concluded that the NaCMC/CS/n-HA spiral-cylindrical scaffolds have a high potential for the treatment of large bone defects and could also be effective in the healing of critical-sized segmental bone defects [92].

### 2.3. Wound Healing

**Figure 10:**
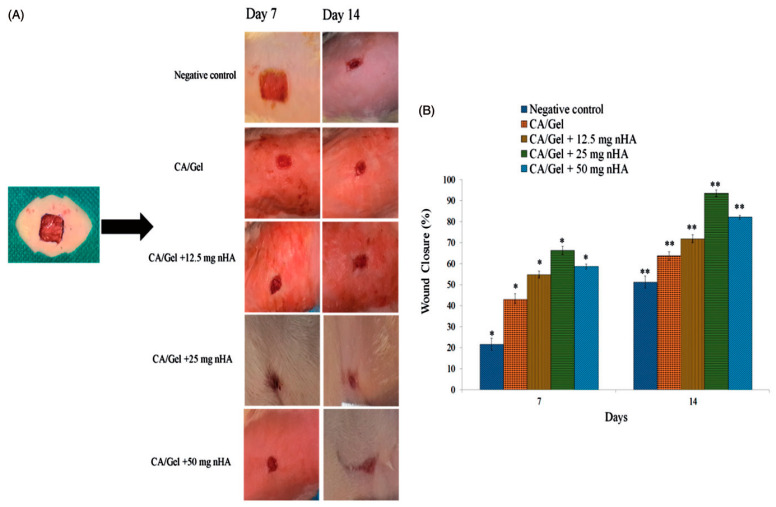
Cosmetic appearance of the wounds treated with the cellulose acetate/gelatin/nano-hydroxapatite dressings 7 and 14 days post-wounding (**A**) and histograms comparing the wound closure percentages at the end of the 7th and 14th day post-wounding (**B**) (reproduced with permission from Ref. [50]). * Significant difference from all in day 7 (*p* < 0.5). ** Significant difference from all in day 14 (*p* < 0.5).

Multiple studies emphasized the important role of calcium in the normal homeostasis of the skin and keratinocyte proliferation and differentiation [93]. Nano-hydroxyapatite was used as a calcium source and incorporated in cellulose acetate/gelatin solutions. The composite membranes were prepared by electrospinning and their potential as wound healing mats was evaluated via a full-thickness excision wound model in healthy adult male Wistar rats. The results showed that the concentration of nanoparticles was in direct correlation with porosity, hydrophilicity, water vapor transmission rate, and cellular proliferation. The synthesized dressings had higher wound closure percentages compared to the sterile gauze used as control and also improved collagen synthesis, re-epithelialization, neovascularization and the overall cosmetic appearance of the wounds (Figure 10). Best results were obtained for the cellulose acetate/gelatin membranes with a hydroxyapatite content of 25 mg/10 cc of polymer solution, a higher inorganic particles percentage leading to hyperplasia of the epidermal layer and foreign body reactions [50].

### 2.4. Controlled Drug Delivery

Drug delivery systems are gaining momentum in the biomedical field due to their advantages such as controlled release of the therapeutic substance, which ensure the maintenance of its level within a desired range and the need for fewer administrations associated with an increased patient comfort [94]. Nystatin (Nys), a polyene antibiotic obtained from *Streptomyces noursei* bacterial strain, was used as model drug to evaluate the controlled drug delivery ability of cellulose acetate/hydroxyapatite membranes. The composites were obtained by adding calculated amounts of hydroxyapatite and nystatin in the cellulose acetate solution (CA/Nys mass ratio ~3.34), followed by casting on Petri plates and solvent evaporation [55]. The release rate of nystatin was higher when hydroxyapatite was present in the membranes. The release enhancement was associated to the increased membrane porosity generated by the addition of HA. The connection between membrane porosity and the drug release rate was also observed by Wang et al. that prepared CA membranes for the transdermal delivery of scopolamine using PEG 1000 as pore-forming agent. The scopolamine release was found to be proportional to the PEG content, a higher PEG percentage generating a more porous structure and higher release rates respectively [95]. Another important aspect that influenced the Nys release was related to the retention of drug molecules by the hydroxyapatite crystals formed on the membrane surface, thus favoring the drug dispersion and also increasing its exposure to the dissolution media during the release tests. Hydroxyapatite and nystatin were also observed in the cross-section SEM images as white spots spread over the internal surface of the membrane, therefore the particles were not retained entirely on the surface and a part of them were incorporated in the polymeric matrix. The CA membranes achieved a sustained Nys release for up to 50 h, the CA/HA ones releasing the drug faster. According to literature, they can be characterized as an effective controlled drug delivery device because the drug release occurred over a period of time greater than 24 h [96]. Chitosan (CS) [97] and polycaprolactone (PCL) [98] were also investigated as controlled delivery devices for nystatin in the form of films and capsules. However, the CS films maintained a gradual nystatin release for only 5 h, reaching a plateau phase in approximately 1–2 h while the PCL nanocapsules completely released the drug after 9 h. Consequently, CA/HA-based delivery systems are a viable option for applications where a prolonged drug delivery is required.

### 2.5. Hemodialysis

Hemodialysis is the process of removal of waste and extra water from blood using an external filter called a dialyzer, which contains a semipermeable membrane [99]. Cellulose acetate was among the first polymers used for the production of hemodialysis membranes, the renewability, biodegradability, and biocompatible nature as well as low cost and good toughness recommending it for this application [100]. Due to the binding sites on the particles surface, hydroxyapatite can effectively retain biomolecules. This aspect is very important because in hemodialysis the toxins should be removed and useful biomolecules like proteins and carbohydrates should be retained. Hayder et al. investigated the effect of hydroxyapatite on the morphology, water flux, urea clearance, and glucose and bovine serum albumin (BSA) retention of cellulose acetate/polyethylene glycol membranes prepared by phase inversion. After the addition of hydroxyapatite, macro-pores were formed in the compact cellulose acetate matrix due to the leech out of filler particles. The porosity and increased hydrophilicity of the composite membranes determined a significant increase in water and urea permeation, the calculated permeability of the composites membranes being 0.075 L/h m bar for water and 0.035 L/h m bar for urea compared to 0.005 and 0.0037 L/h m bar in the case of pure CA. However, only low quantities of glucose and bovine serum albumin passed through the membranes [51]. These results, together with the reported hemocompatibility of cellulose acetate [101], show promising prospects for the use of hydroxyapatite as functional filler in hemodialysis membranes.

## 3. Conclusions and Future Perspectives

The present review was focused on the systematical presentation of the preparation methods and functional applications of cellulose derivatives/hydroxyapatite composite membranes. The manufacturing techniques varied from phase inversion by immersion precipitation or solvent evaporation to electrospinning and gradual electrostatic assembly, the optimal method being selected depending on the type of cellulose derivative used as an organic matrix. For example, cellulose acetate was found to be appropriate especially for electrospinning and phase inversion while the anionic nature of carboxymethylcellulose recommended it for gradual electrostatic assembly in combination with oppositely charged polymers. The reviewed studies showed that the addition of hydroxyapatite can impart desired characteristics to the surface and structure of the membrane thus improving its performance. However, an important issue is represented by the aggregation tendency of the hydroxyapatite particles especially when phase inversion is used for membrane preparation. Various strategies were employed to obtain a homogenous filler dispersion, the addition of surfactants, plasma modification of the inorganic particles, and ultrasound assisted mixing showing promising results. Regarding the application fields of the hybrid cellulose derivatives/hydroxyapatite membranes, water purification, hemodialysis, and tissue engineering are among the domains intensively researched.

Future perspectives are mainly related to the biomedical applications of composite membranes with hydroxyapatite, particularly osseointegration combined with the controlled release of therapeutic agents. The synthesis of hydroxyapatite with controlled particle dimensions to favor an optimal osseointegration represents one of the challenges of the near future. Too large dimensions hinder the development of pre-osteoblasts, while too small ones are characterized by an inefficient activity of these particles. Another future direction is represented by the obtainement of hydroxyapatite from natural sources, other than bone (e.g., snail shells or seashells) as well as its wide scale synthesis from anorganic precursors. A domain that will know an explosive development will be the one of controlled release of pharmaceutically active substances from hydroxyapatite particles, antibiotics and cytostatics being the main classes of drugs that will be studied for such applications. In the dental field, composite membranes are already used to favor osseointegration. The capacity of the membrane to release antibiotics, locally, from particles of hydroxyapatite will enhance both quality and efficiency of the medical act and will also improve the patient’s life. The utilization of composite membranes with hydroxyapatite for such applications will provide multiple simultaneous advantages—the membrane will favor implant osseointegration in the case of major bone defects generated by the extraction of the tumoral mass and the targeted release of cytostatics will be associated with an augmented treatment efficiency and with the minimization of the toxic impact of these drugs on the human body.

Principle practical gaps are related to the control of porosity for both components—hydroxyapatite and the membrane. New methods for hydroxyapatite production will be accompanied by processing steps like controlled milling or sintering in order to provide the desired porosity for the required application. In the domain of membranes the porosity can be easily controlled, but a major challenge will be the synthesis of membranes with macroporosity and nanoparticles in their structure. Most probably, the easiest way to address this problem will be the chemical bonding between hydroxyapatite and the cellulose derivative, which will remain a future challenge.
